# A natural language processing approach towards harmonisation of European medicinal product information

**DOI:** 10.1371/journal.pone.0275386

**Published:** 2022-10-20

**Authors:** Erik Bergman, Kim Sherwood, Markus Forslund, Peter Arlett, Gabriel Westman

**Affiliations:** 1 Swedish Medical Products Agency, Uppsala, Sweden; 2 European Medicines Agency, Amsterdam, Netherlands; 3 Department of Medical Sciences, Uppsala University, Uppsala, Sweden; STL UMR8163 CNRS, FRANCE

## Abstract

Product information (PI) is a vital part of any medicinal product approved for use within the European Union and consists of a summary of products characteristics (SmPC) for healthcare professionals and package leaflet (PL) for patients, together with the product packaging. In this study, based on the English corpus of the EMA product information documents for all centrally approved medicinal products within the EU, a BERT sentence embedding model was used together with clustering and dimensional reduction techniques to identify sentence similarity clusters that could be candidates for standardization. A total of 1258 medicinal products were included in the study. From these, a total of 783 K sentences were extracted from SmPC and PL documents which were aggregated into a total of 284 and 129 semantic similarity clusters, respectively. The spread distribution among clusters shows separation into different cluster types. Examples of clusters with low spread include those with identical word embeddings due to current standardization, such as section headings and standard phrases. Others show minor linguistic variations, while the group with the largest variability contains variable wording but with significant semantic overlap. The sentence clusters identified could serve as candidates for further standardization of the PI. Moving from free text human wording to auto-generated text elements based on multiple-choice input for appropriate parts of the package leaflet and summary of product characteristics, could reduce both time and complexity for applicants as well as regulators, and ultimately provide patients and prescribers with documents that are easier to understand and better adapted for search availabilities.

## Introduction

The product information (PI) is a vital part of any medicinal product approved for use within the European Union. It consists of the summary of products characteristics (SmPC) for healthcare professionals and package leaflet (PL) for patients, product packaging as well as an Annex which sets out conditions and restrictions for supply and the safe and effective use of the medicinal product. These documents, created from templates, explain how the product should be used and describe the expected benefits and risks associated with its use [1–3].

In 2020, following discussions with stakeholders, the European Medicines Agency presented key principles outlining a harmonized approach to develop and use an openly accessible and forward-compatible electronic format for product information (ePI) for human medicines across the EU. The ePI project mainly concerns the technical aspects of documents and aims to enhance accessibility of PI and promote best practices for creating product information for medicinal products [4]. Developing an electronic format with structured elements accommodates benefits brought by continuously evolving digital opportunities which will enable more efficient retrieval of information and facilitate the use of alternative e-platforms. As ePI can be read by machines, ePI information can flow to other systems such as electronic health records and e-prescribing systems. This will hopefully facilitate targeted delivery of the right information to the right user at the point of need.

In addition to the technical aspects, there will be challenges remaining after the expected output from the ePI project has been delivered. Several of these are related to the language itself contained in the documents, which are created by applicants and regulators in an iterative process to obtain a marketing authorization. Although there are current linguistic PI standards implemented manually for certain aspects of style, terminology and use of abbreviations, substantial variability is introduced between medicinal products in sentences where identical messages could be communicated. This process is time-consuming, may cause difficulties in search functions, creates uncertainty as to whether the content of different sets of PI is identical, and could potentially result in an increased risk of medication error [5, 6]. Whereas standardised messages could and should be used already today, a digitalization of the format will facilitate the use of exact standards, as well as to offer possibilities to streamline, simplify and speed up the regulatory processes.

The field of natural language processing (NLP) has been rapidly evolving since the introduction of transformer models [7]. These models are, without supervision or human imputation, trained on huge corpuses of text and contains high-dimensional semantic word embeddings based on textual context. In 2018, Devlin et al presented BERT, a *Bi-directional Encoder Representation from Transformers* model with 340 million parameters, which became state of the art in the field of NLP and has found many applications and undergone adaption to specific tasks [8]. Lately, BERT has been outperformed—particularly in creative NLP applications—by the much larger generative language models such as GPT-3 which have hundreds of billions of parameters [9]. However, the BERT architecture has remained a well-performing alternative for tasks such as classification and needs substantially less computing power.

NLP techniques have previously been applied to extract information from FDA medicinal product information [10]. Also, there is previous work on model-based standardisation of clinical information and lexical simplification of technical terms [11–13]. In this study, based on the English corpus of the EMA PI documents for all centrally approved medicinal products within the EU, we use a BERT sentence embedding model together with clustering and dimensional reductional techniques to identify PI sentence similarities that could be standardized, for the benefit of patients, prescribers, and marketing authorization holders alike.

## Materials and methods

### Text acquisition and pre-processing

The text corpus was compiled on May 3, 2022, by scripted downloading of all available English language PI files for all centrally approved medicinal products within the EU, from the EMA website. PL and SmPC documents for each medicinal product, excluding multiplicate documents for medicinal products with more than one strength or pharmaceutical preparation, were used. The PDF files were scraped using the *pdfplumber* version 0.6.1 package in *Python* 3.8.10 to extract all text except page numbering, headers, and footers.

**Fig 1:**
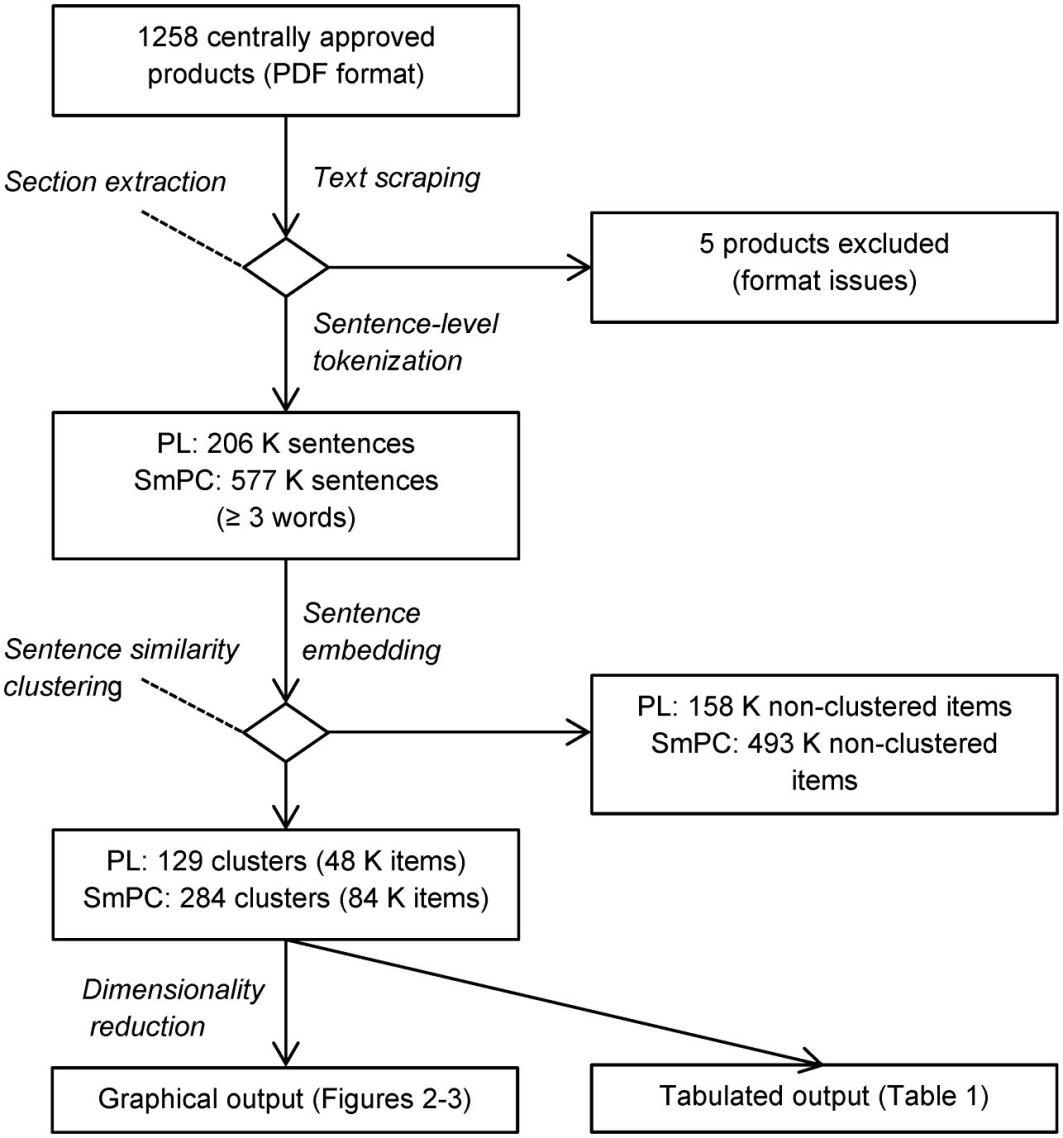
Description of the data processing pipeline.

Line breaks and special characters (excluding punctuation characters) were removed, and punctuation was added to sentences where this was missing (such as headings) to avoid false aggregation. All paragraphs were tokenized on a sentence level using the *NLTK* version 3.7 tokenizer and filtered to exclude sentences shorter than three words. The complete data processing pipeline is shown in Fig 1.

All sentences were embedded, i.e., transformed into a 768-dimensional output vector, using the *pre-trained SBERT model all-mpnet-base-v2*. Detailed information about the model is available in the model card [14].

### Clustering and dimensionality reduction

*DBSCAN* from *scikit-learn* version 1.0.2 with ε = 0.45 and a minimum cluster size of 50 was used to aggregate adjacent sentence embeddings into similarity clusters within the full embedding space. For visualization purposes, projection algorithms were explored to reduce the high-dimensional space to a flat 2-d projection. Due to the high dimensionality of the data and expected shape of clusters, t-SNE from [15] *scikit-learn version 1*.*0*.*2* was chosen over *UMAP* from *umap-learn version 0*.*5*.*3* [16]. t-SNE was applied using principal component analysis initialization, was run for 500 iterations with a perplexity of 20, learning rate of 200 and random state 23. To allow further analysis of cluster shape and spread, cluster centroids were calculated using *K-means* from *scikit-learn version 1*.*0*.*2*.

## Results

A total of 1258 medicinal products were initially included in the study, of which 5 were subsequently excluded due to document compatibility issues. From these, a total of 783 K sentences were extracted from PL and SmPC documents. The length and distribution of sentences among subsections is illustrated in S1 File.

**Fig 2:**
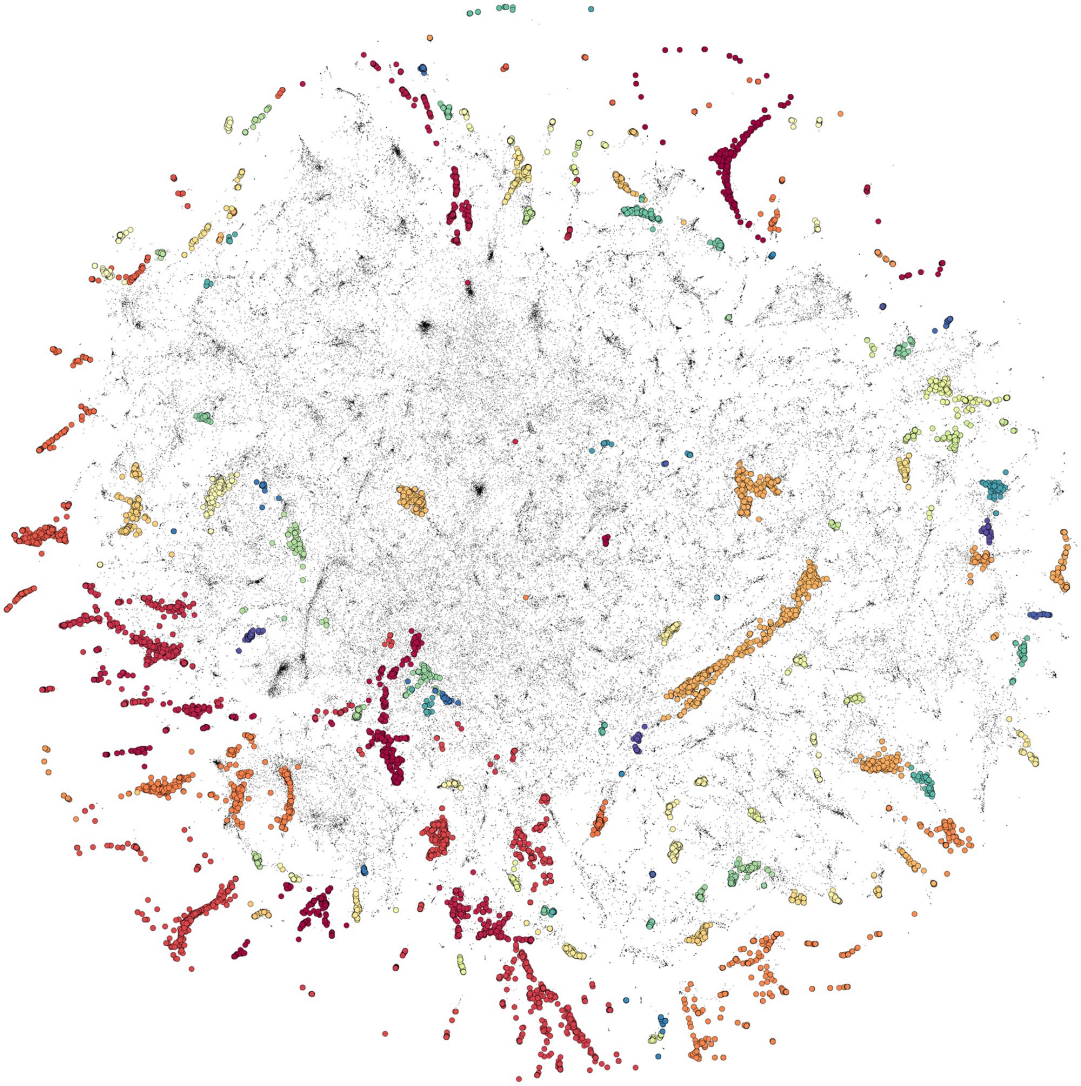
t-SNE projection of 206 K package leaflet sentence embeddings, showing 129 clusters in colour coding and non-clustered sentences in black.

**Fig 3:**
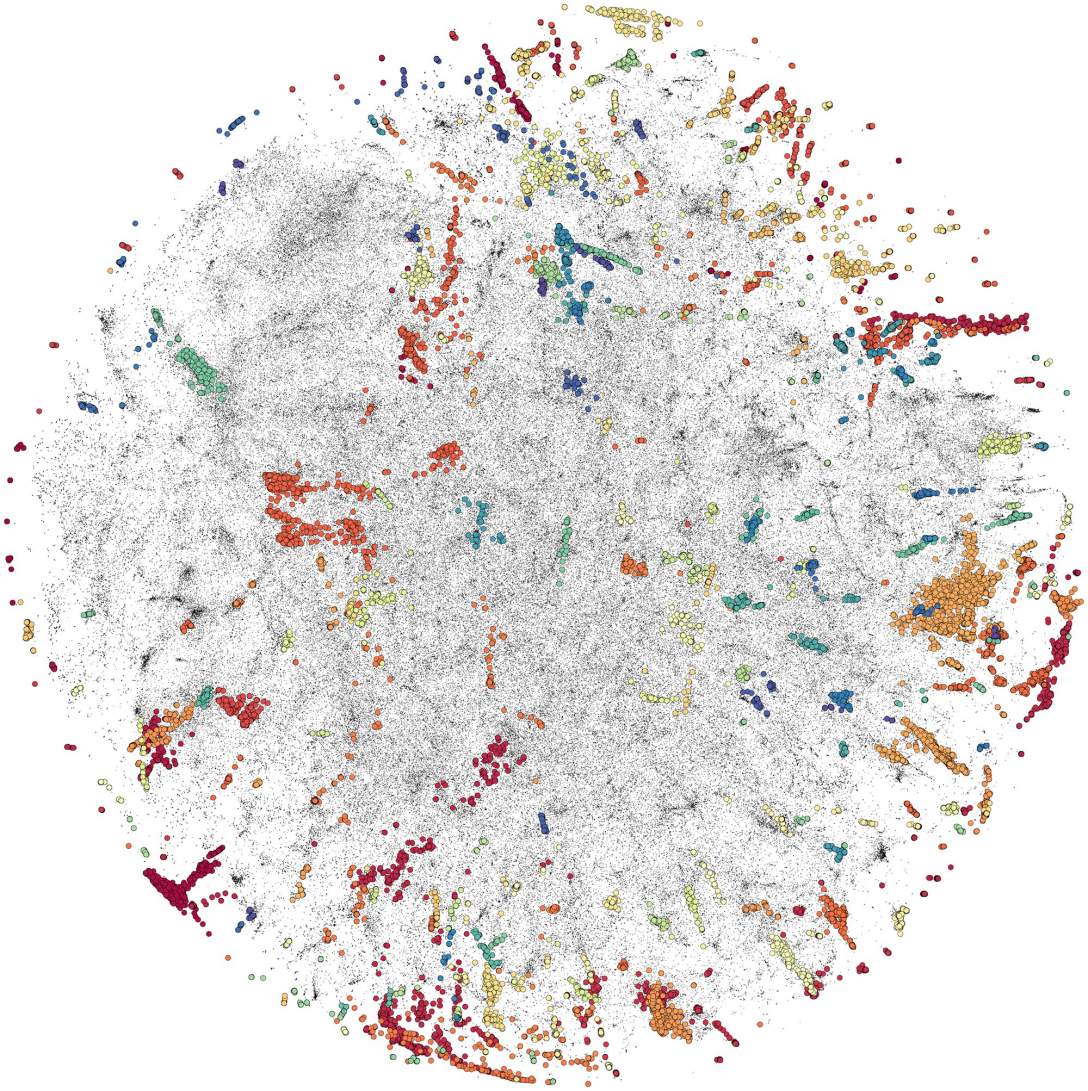
t-SNE projection of 577 K summary of product characteristics sentence embeddings, showing 284 clusters in colour coding and non-clustered items in black.

From the representations in full embedding space, PL and SmPC sentences were analysed separately, generating a total of 129 and 284 similarity clusters, respectively (Figs 2 and 3).

**Fig 4:**
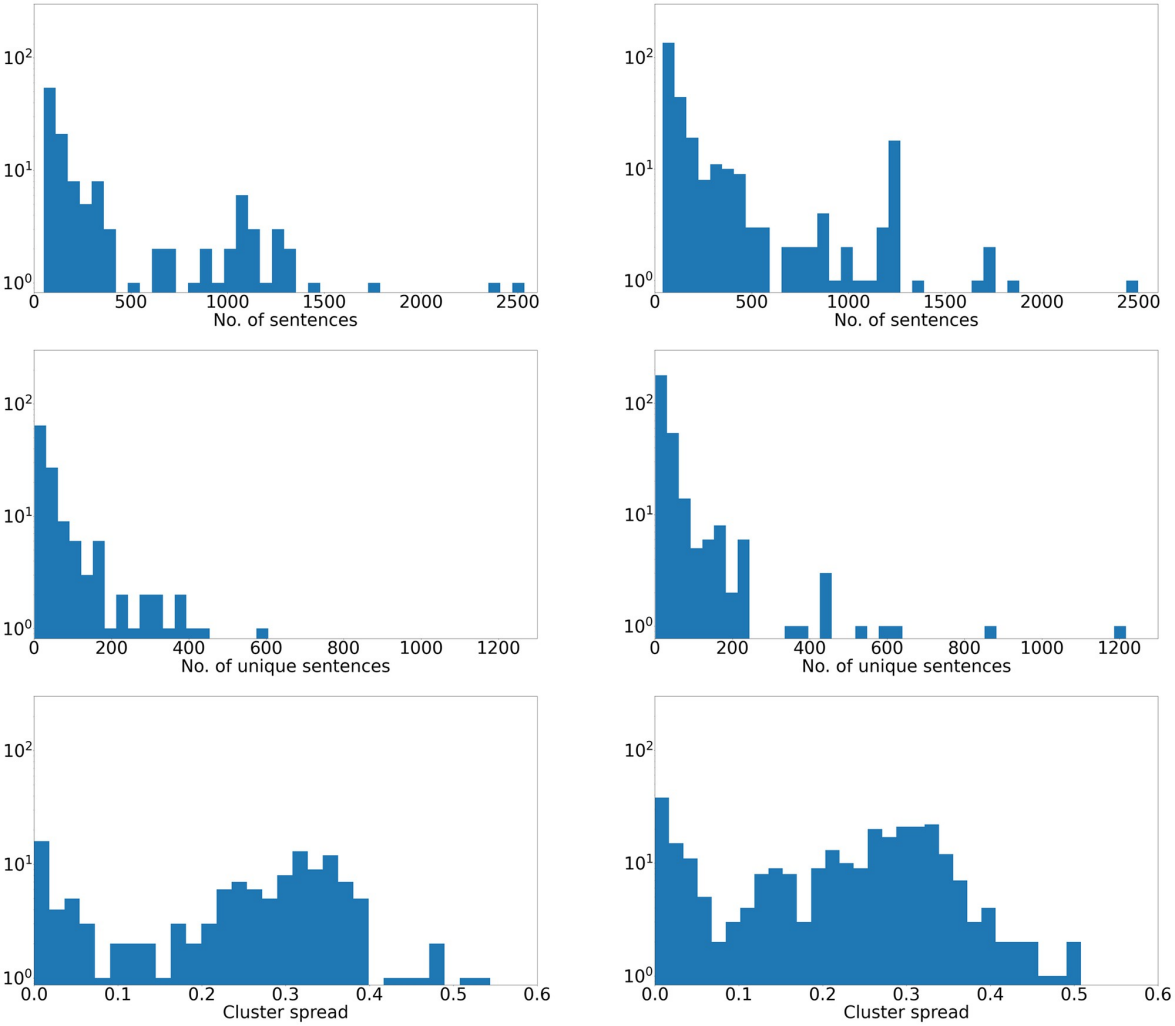
Cluster characteristics for PL (left) and SmPC documents (right). Distribution of total cluster size (top) and number of unique sentences (middle). Cluster spread histogram (bottom) shows mean Euclidean distance from cluster centroid, and suggests separation into different cluster types with regards to variability.

Although the mean Euclidian distances used to estimate the spread of individual clusters in space should be analysed with caution, as interpretability can decrease with increasing dimensionality, the distribution among clusters (Fig 4) indicates separation into different cluster types depending on the level of linguistic variability. Examples with low spread—i.e., low variability—include those with identical embedding due to current standardization such as section headings and standard phrases. Others show minor linguistic variations, while the group with the largest variability contains variable wording but with significant semantic overlap at least on a thematic level. Examples from a range of categories with the corresponding cluster characteristics, are presented in Table 1.

**Table 1:** Examples of sentence clusters within the PL and SmPC language space. Up to ten variants listed per cluster.

**Package leaflet**			
**Cluster id**	**Spread**	**Number of sentences**	**Number of unique sentences**
113	0,026	52	2
(n = 47) During HIV therapy there may be an increase in weight and in levels of blood lipids and glucose. (n = 5) During treatment for HIV there may be an increase in weight and in levels of blood lipids and glucose.			
**Cluster id**	**Spread**	**Number of sentences**	**Number of unique sentences**
15	0,31	1750	320
(n = 528) If you get any side effects, talk to your doctor or pharmacist. (n = 347) If you get any side effects, talk to your doctor, pharmacist or nurse. (n = 91) If you get any side effects, talk to your doctor or nurse. (n = 85) If you get any side effects, talk to your doctor. (n = 31) If you get any side effects, talk to your doctor, pharmacist, or nurse. (n = 20) If you get any side effects, talk to your doctor or pharmacist or nurse. (n = 15) If you get any side effects talk to your doctor, pharmacist or nurse. (n = 14) If you get any side effects talk to your doctor or pharmacist. (n = 14) If you get any side effects, talk to your doctor, nurse or pharmacist. (n = 12) Tell your doctor, pharmacist or nurse if you notice any of the following side effects:.			
**Cluster id**	**Spread**	**Number of sentences**	**Number of unique sentences**
10	0,46	1316	425
(n = 281) Do not take a double dose to make up for a forgotten dose. (n = 89) Do not take a double dose to make up for a forgotten tablet. (n = 34) Do not use a double dose to make up for a forgotten dose. (n = 24) If you miss a dose, take it as soon as you remember. (n = 18) If you forget to take a dose, take it as soon as you remember. (n = 17) Do not inject a double dose to make up for a forgotten dose. (n = 16) Then take your next dose at the usual time. (n = 15) Do not take a double dose to make up for forgotten individual doses. (n = 15) Then take the next dose as usual. (n = 15) If you forget for more than 12 hours, simply take the next single dose at the usual time.			
**Summary of product characteristics**			
**Cluster id**	**Spread**	**Number of sentences**	**Number of unique sentences**
201	0	52	1
(n = 52) Lipid disorders should be managed as clinically appropriate.			
**Cluster id**	**Spread**	**Number of sentences**	**Number of unique sentences**
109	0,15	92	20
(n = 34) If not used immediately, in-use storage times and conditions are the responsibility of the user. (n = 27) If not used immediately, in-use storage times and conditions prior to use are the responsibility of the user. (n = 6) Other in-use storage times and conditions are the responsibility of the user. (n = 4) Other in-use storage times and conditions are under the responsibility of the user. (n = 3) If not used immediately, in-use storage times and conditions are the responsibility of user. (n = 2) If not used immediately, in use storage times and conditions prior to use are the responsibility of the user. (n = 2) If not used immediately, in- use storage times and conditions prior to use are the responsibility of the user. (n = 2) In-use storage times and conditions prior to use are the responsibility of the user. (n = 1) If not used immediately, in-use storage times and conditions are the responsibility of the user and should. (n = 1) If not used immediately, in-use storage times and conditions prior to use are the responsibility of the user and.			
**Cluster id**	**Spread**	**Number of sentences**	**Number of unique sentences**
73	0,31	77	49
(n = 8) Women of childbearing potential have to use effective contraception during (and up to 6 months after) treatment. (n = 4) Women of childbearing potential have to use effective contraception during treatment. (n = 4) Women of childbearing potential have to use effective contraception during and up to 2 years after treatment (see waiting period below) or up to 11 days after treatment (see abbreviated washout period below). (n = 4) Women of childbearing potential and men have to use effective contraception during and up to 3 months after treatment. (n = 4) Women of childbearing potential have to use effective contraception during and up to 6 months after treatment. (n = 4) Women of childbearing potential must use effective contraception during treatment. (n = 4) Women of childbearing potential have to use effective contraception during treatment and at least up to 21 days after treatment discontinuation (based on pitolisant/metabolites half-life). (n = 2) Women of childbearing potential should use an effective method of contraception during treatment and for at least 17 weeks after treatment. (n = 2) Women of childbearing potential should use effective contraception during treatment. (n = 2) Women of childbearing potential must use effective contraception during treatment and 3 months thereafter, and immediately inform the treating physician if a pregnancy occurs (see section 5.3).			
**Cluster id**	**Spread**	**Number of sentences**	**Number of unique sentences**
50	0,5	1739	873
(n = 89) No dose adjustment is required. (n = 40) No dose adjustment is necessary. (n = 37) No dose adjustment is required (see section 5.2). (n = 20) No dose adjustment is required based on age. (n = 16) No dose adjustment is required in patients with mild or moderate hepatic impairment. (n = 16) No dose adjustment is required in patients with renal impairment (see section 5.2). (n = 12) No dose adjustment is recommended. (n = 11) No dosage adjustment is required (see section 5.2). (n = 10) Dose adjustment is not required. (n = 10) No dose adjustment is needed in patients with mild to moderate hepatic impairment.			

## Discussion

Natural language processing holds great promise to generate value in the field of pharmaceutical regulatory science, including both drug development and formal regulatory processes. Here, we illustrate a relatively straight-forward approach to create meaningful sentence-level numerical representations of the nearly one million sentences from all EU centrally approved SmPC and PL documents, to facilitate both future standardization as well as NLP research. In addition to our similarity clusters, we provide a freely available database containing a total of 783 K sentence tokens that are indexed and mapped to the medicinal product, document type and document subsection from which they originated.

We chose to analyse SmPC and PL documents separately, in line with the regulatory practise to compile these documents in separate processes tailored for their respective group of professional and non-professional end users. However, given that many messages are shared across the two types of documents, the linguistic relationship and potential for a more unified workflow should be explored in the future.

Although not specifically trained on a corpus of medical or pharmaceutical language, the BERT sentence embeddings allowed for semantic analysis of medicinal product information documents. This could to some extent be a result of regulatory language standards, making these documents contain far less medical specialist terms compared to health records. However, a language model trained on a corpus including medical and regulatory text would likely perform even better.

With the parameters for clustering that were used in this project, a total of 15% of sentences in the SmPCs and 23% of sentences in the PL documents were assigned to a semantic cluster. The absolute level of clustering should be interpreted with caution as it is highly dependent on what level of in-cluster similarity and minimum cluster size is sought, but the relative difference in clustering rate between SmPCs and PLs indicates that the PLs have a lower degree of linguistic variability.

By mapping the complete PI language space for European centrally approved medicinal products, we can show that there is a high level of semantic similarity in substantial parts of the documents where there currently is no fixed standard, or where standards exist but supporting systems should be developed or vocabularies used more frequently.

Semantic similarities may also point out the need for a certain structure for expanded electronical use, where existing standard sentences could be automatically populated or linked via reference data vocabularies. Such format would facilitate the use of existing established standard references, already translated into all EU languages. Where information such as “No dose adjustment is needed/No dose adjustment in children is needed” has been found, it may not need harmonisation as such, but rather electronic structure connected to both standard and subheading, i.e., both wording and place in the document will be important when forming structured standards.

Fields where standardized sentences are of great value, such as storage precautions and certain warning texts have already been harmonised by presenting standard sentences in EMA guidelines. The approach developed in this study could be used for further standardization, potentially valuable for regulatory processes as well as for down-stream use of the product information. Nevertheless, although the sentence clusters identified could serve as candidates for future standardization, the PI will always contain unique product-specific statements.

In the current approach only centrally approved medicinal products were included, which are all fairly new and where a single version of product information is used as a base for translation in all EU member states. Expanding the analysis to products approved in decentralized or national procedures, where documents by procedural nature vary between products of even the same active substance or therapeutic class, could provide further insight and further the potential for standardisation.

## Conclusion

In this study, we have shown that currently available data science tools can identify semantically similar statements in medicinal product information. This could serve as a basis in a future process of standardisation, which would allow automation for parts of the PI compilation process. Moving from free text human wording to auto-generated text based on multiple-choice input for appropriate parts of the summary of product characteristics and package leaflet would reduce both time and complexity for applicants and regulators, and ultimately provide patients and prescribers with documents that are easier to understand and better adapted for search availabilities. For the foreseeable future, it will remain essential that the final documents are assessed by domain experts at the competent authorities involved, keeping the human in the loop throughout the process.

## Supporting information

S1 File Product information document characteristics. (PDF)
