# Supplementary material for: A natural language processing approach towards harmonisation of European medicinal product information
Source: PLoS One. 2022 Oct 20;17(10):e0275386. doi: 10.1371/journal.pone.0275386 (PMC9584511; doi:10.1371/journal.pone.0275386)
Supplement: S1 File — (PDF) [file pone.0275386.s001.pdf]

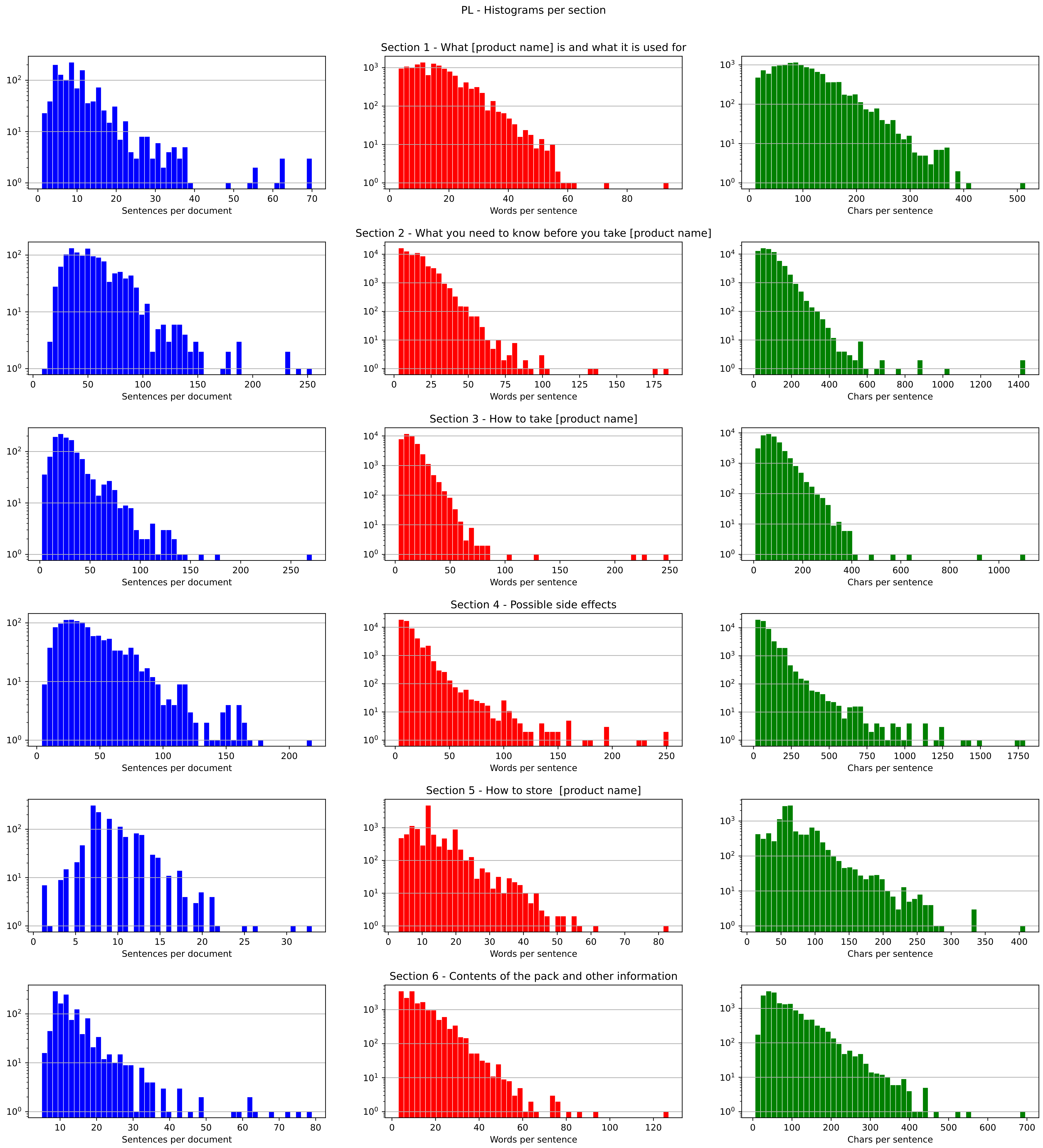

PL - Histograms per section
Section 1 - What [product name] is and what it is used for
Section 2 - What you need to know before you take [product name]
Section 3 - How to take [product name]
Section 4 - Possible side effects
Section 5 - How to store [product name]
Section 6 - Contents of the pack and other information
Sentences per document
Words per sentence
Chars per sentence

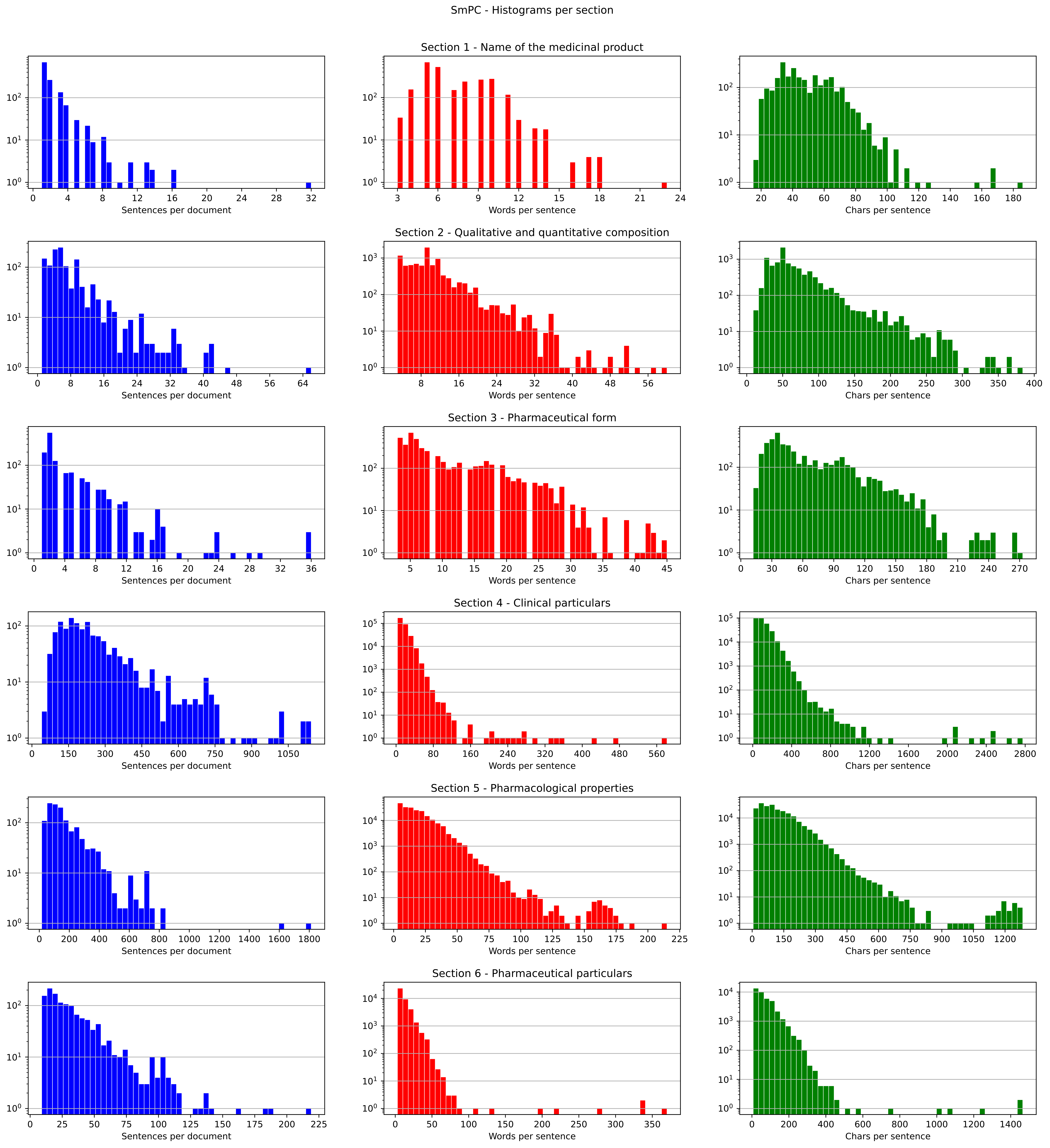
SmPC - Histograms per section
Section 1 - Name of the medicinal product
Section 2 - Qualitative and quantitative composition
Section 3 - Pharmaceutical form
Section 4 - Clinical particulars
Section 5 - Pharmacological properties
Section 6 - Pharmaceutical particulars
Sentences per document
Words per sentence
Chars per sentence
